# Effect of vaporized Cannabidiol (CBD) on neuropathic pain and its potential implication for development of chronic lung inflammation in rats

**DOI:** 10.1186/s42238-026-00459-z

**Published:** 2026-06-19

**Authors:** Priyadarshini Dutta, Ignacio Javier Novoa Cornejo, Vijaya Prakash Krishnan Muthaiah, Ravikumar Aalinkeel, Tracey A. Ignatowski, Supriya D. Mahajan

**Affiliations:** 1 Department of Medicine, Division of Allergy, Immunology, and Rheumatology, Jacobs School of Medicine and Biomedical Sciences, Clinical Translational Research Center, University at Buffalo, The State University of New York, Buffalo, NY 14203 USA; 2 Department of Pathology and Anatomical Sciences, Jacobs School of Medicine and Biomedical Sciences, University at Buffalo, The State University of New York, Room 4256, 955 Main Street, Buffalo, NY 14203 USA; 3 Neuroscience Program, Jacobs School of Medicine and Biomedical Sciences, University at Buffalo, The State University of New York, 955 Main Street, Buffalo, NY 14203 USA; 4 Department of Rehabilitation Science, School of Public Health and Health Professions, University at Buffalo, The State University of New York, 633 Kimball Tower, Buffalo, NY 14214 USA

**Keywords:** Cannabidiol, Neuropathic Pain, Vaping, E-Cigarettes, Analgesia, Lung Inflammation

## Abstract

**Background:**

Cannabidiol (CBD) use in e-cigarettes is increasing, and its vaping involves using CBD in conjunction with other chemical additives such as propylene glycol (PG), vegetable glycerin (VG) and food flavorings, which help vaporize the CBD and enhance the vaping experience. Chronic neuropathic pain is challenging to treat with current medications, and there is growing interest in the use of medical cannabis for this condition. While the effects of vaping CBD could be beneficial for people with neuropathic pain, the potential detrimental effects on the lungs after continued exposure are not fully known. Whereas acute respiratory failure has been associated with vaping Cannabis, this study was undertaken to assess effects of CBD vaping on lung pathology and analgesic effectiveness in rats with sciatic nerve chronic constriction injury and examine potential mechanisms underlying the pathophysiology associated with CBD vaping.

**Methods:**

We used an in-house designed and assembled prototype of an automated vaping exposure system that enables precise quantitation of vaping for *in-vivo* exposure of rats with neuropathic pain.

**Results:**

Our results showed that CBD vapor exposure significantly alleviated pain, and modulation of cytokines and the NLRP3 (NOD-like receptor family, pyrin domain-containing 3) inflammasome pathway may underlie the CBD-induced effects in lung tissue, thereby affecting lung pathophysiology. Further, exposure to chemical additives like PGVG (Propylene Glycol and Vegetable Glycerin) resulted in a substantial inflammatory response indicating that these substances are by no means safe additives in e-cigarettes.

**Conclusions:**

Thus, while CBD vaping may be beneficial in the management of neuropathic pain, it may cause substantial lung injury by promoting significant inflammatory changes in the lung.

## Highlights

- This article presents valuable insights into the potential risks associated with CBD vaping.
- The dual nature of CBD vapor as a treatment for chronic pain and danger to lung health is reported.
- These findings could potentially help clinicians with early identification of pain patients self-medicating with CBD vaping who are experiencing respiratory distress and to choose alternative analgesic strategies.

## Background

Inhalation of vaporized cannabis extracts via electronic cigarette devices for chronic pain or recreational use is the most common cannabis consumption route (Giroud et al. 2015). Cannabidiol (CBD), a cannabinoid from Cannabis sativa, is one of over 100 identified cannabinoids. Alongside tetrahydrocannabinol (THC) and cannabinol, CBD has gained popularity for its health benefits. CBD has shown anti-inflammatory, anti-proliferative, apoptotic, analgesic, and anti-epileptic properties (Calapai et al. 2020; Milando and Friedman 2019; Vučković et al. 2018). Our research focuses on CBD due to its non-psychoactive effects.

Cannabinoids can be beneficial in treating a range of diseases, including neuropathic pain. Neuropathic pain has been defined by the International Association for the Study of Pain (IASP) as “pain caused by a lesion or disease of the somatosensory nervous system” and is a type of chronic pain lasting for more than three months (Murnion 2018). This condition affects 8–10% of U.S. adults, with one in five experiencing chronic pain, and one in 14 enduring “high-impact” chronic pain (Dowell et al. 2022). Neuropathic pain is challenging to treat with current medications, prompting growing interest in medical cannabis.

Furthermore, cannabis inhalation reduced self-reported pain by 42–49% (Cuttler et al. 2022); women reported higher baseline and post-cannabis pain than men, but men experienced greater pain reductions (Cuttler et al. 2022). Interestingly, analysis showed no correlation between CBD concentration and reductions in self-reported pain severity, suggesting a complex relationship between CBD and pain relief. Further research is needed to clarify the mechanisms underlying CBD's analgesic effects and interactions with other cannabinoids. Interestingly, vaping was associated with greater reductions in joint pain compared to smoking, and lower doses were linked to more significant reductions in nerve pain.

Several studies support the safety and efficacy of short-term, low-dose cannabis vaporization for neuropathic pain treatment (Giroud et al. 2015; Hamberger and Halpern-Felsher 2020; Pichelstorfer et al. 2016). E-cigarette liquids marketed as containing only CBD have become more widely available (Peace et al. 2016), though some may contain other psychoactive drugs (Poklis et al. 2019). Many vaping products, used for inhaling Cannabis/CBD/THC, include harmful additives like Vitamin E Acetate, Diacetyl, Formaldehyde, vegetable glycerin (VG), and various flavorings, none of which are regulated by the FDA (Merecz-Sadowska et al. 2020). Vaporizing cannabis has become the preferred method for medical use, as it eliminates smoke, reducing exposure to toxic particulates and the characteristic odor of cannabis (Sosnowski and Kramek-Romanowska 2016). The CDC reported that e-cigarette or vaping products with cannabis additives were linked to 84% of lung injury cases (Centers for Disease Control and Prevention (CDC), 2019). E-cigarette use-associated lung injury (EVALI) is marked by lung inflammation, which correlates with exposure to toxic substances in e-cigarette liquids and the frequency and duration of exposure (Aberegg et al. 2020; Antoniewicz et al. 2019; Siegel et al. 2019).

Pre-clinical experiments often use intraperitoneal (i.p.) or subcutaneous (s.c.) CBD injections in rodents, which may not reflect the pharmacokinetic profile from vapor inhalation. Rats given CBD vapor inhalation achieved concentration-related plasma CBD levels similar to those obtained by injection. In male rats a modest, concentration-dependent reduction in body temperature was observed, which is consistent with physiological effects associated with vapor inhalation rather than a direct thermoregulatory action of CBD. Importantly, nociceptive sensitivity was not markedly altered after CBD vapor exposure (Javadi-Paydar et al. 2019). Collectively, these findings demonstrate that the vapor-inhalation paradigm replicates the pharmacokinetic and physiological outcomes of human inhalation use, thereby supporting its value as a translational preclinical model for investigating the effects of inhaled cannabinoids. Female rats were used to examine the effects of inhaled cannabis vapor with a high concentration of CBD on opioid reward and modulation of nociception (Rivera-Garcia et al. 2023). The findings suggest that inhaling vapor with high levels of CBD modestly relieved neuropathy-induced cold allodynia, without causing adverse effects on lung function, cognitive function, or social behavior (Rivera-Garcia et al. 2023).

CBD vaping via E-cigarettes has surged, particularly among adolescents (Breitbarth et al. 2018; Cullen et al. 2018; Kowitt et al. 2019; Seaman et al. 2020; Traboulsi et al. 2020). However, despite increasing reports in human and clinical contexts, relatively few controlled laboratory studies have examined the mechanistic and physiological effects of CBD vapor exposure. Such preclinical data is essential to complement clinical findings and to better understand the biological impact of inhaled cannabinoids (Boggs et al. 2018). This study aimed to determine the pathologic lung effects of rats exposed to CBD vapor for analgesia to highlight the role of CBD vaping in the development of chronic lung inflammation. Because inflammasome signaling is a key regulator of pulmonary immune responses and inflammation-driven tissue injury, lung pathophysiology was explored by assessing inflammasome activation and inflammatory mediators. Inflammasome activation plays a central role in orchestrating pulmonary inflammatory responses and has also been implicated in e-cigarette–induced lung injury (Tsai et al. 2020; Hosseini et al. 2022). Therefore, this study specifically examined inflammasome signaling and related cytokine mediators to better understand CBD vapor–associated lung pathophysiology. Activation of components such as NLRP3 and the release of cytokines including IL-6 and TNF-α are well-recognized contributors to epithelial disruption, airway inflammation, and reduced lung function following inhalational exposures (Madison et al. 2019; Tsai et al. 2020; Hosseini et al. 2022). Importantly, the potential for CBD vaping to alleviate neuropathic pain was assessed.

## Methods

### Experimental paradigm

A protocol including the research question, key design features, and analysis plan was formulated prior to initiation of this unregistered study. A total of 19 male, Sprague–Dawley rats were used in this study, with two lost during anesthesia induction (ketamine 75 mg/kg, xylazine 10 mg/kg) due to an adverse reaction. Initially, six rats (3 Sham, 3 CCI) were used to learn to perform the chronic constriction injury (CCI) surgery and ensure development of thermal hyperalgesia and allodynia, pain behaviors characteristic of this neuropathic pain model. The remaining 11 rats with CCI were divided into the following exposure groups: CBD vapor (*n* = 5), vehicle (propylene glycol and vegetable glycerin, PGVG) vapor (*n* = 4), and filtered air (*n *= 2). Individual rats were exposed to two weeks (10 days, Monday—Friday) of vapor following CCI surgery and development of pain behaviors as assessed on day-2 post-surgery. Baseline measurements of thermal hyperalgesia, mechanical allodynia, and cold allodynia were taken on Day −4 and Day −1 pre-surgery, with additional thermal hyperalgesia assessments on Day 0 before surgery. Pain measurements were repeated on Day 2 post-surgery for confirmation. Thermal hyperalgesia was measured only to verify successful induction of neuropathic pain after CCI and was not reassessed during vapor exposure, as repeated heat testing can induce sensitization and interfere with ongoing analgesic responses. This approach was intentional, as the primary objective was to evaluate sustained mechanical and cold pain behaviors, which provide more stable and reproducible endpoints for chronic neuropathic pain assessment in rodent models. Instead, post-exposure behavioral evaluations focused on mechanical and cold allodynia, which are more stable indicators of sustained neuropathic pain and treatment effects in this model. Exclusion criteria (established a priori) was absence of pain behaviors on Day-2 post-surgery; there were no exclusions. From Day 6 to Day 17 post-surgery, treatments were administered accordingly. Mechanical allodynia and cold allodynia were reassessed on Day 13 (at 1-week post-vapor exposure and prior to start of week-2 exposure) and Day 17 (at 2 weeks post-vapor exposure) post-surgery, respectively. On Day 21, rats were euthanized, and blood and tissue samples were collected. Body weight was monitored every alternate day throughout the study. A timeline for the experimental paradigm is represented in Schematic 1 below.

**Scheme 1:**
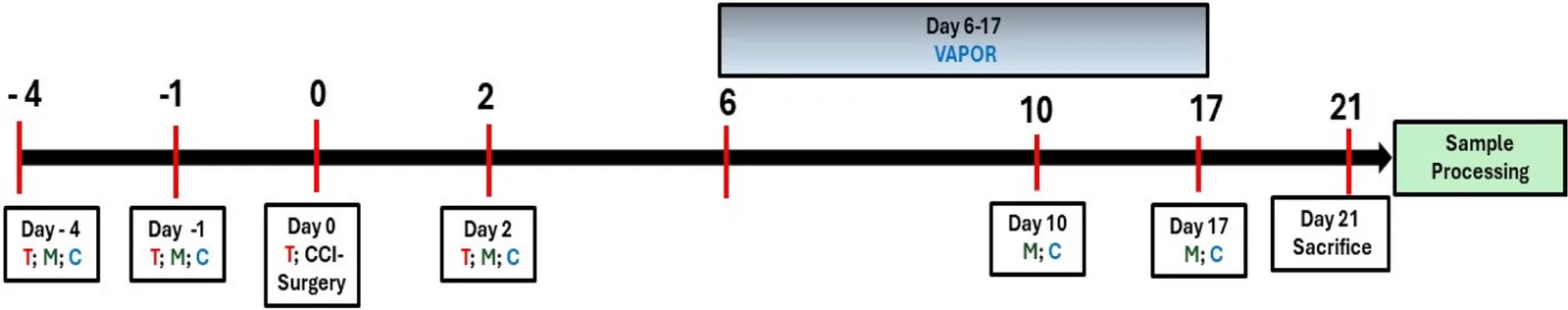
Experimental Paradigm. T- Thermal Hyperalgesia; M- Mechanical Allodynia; C- Cold Allodynia; CCI- chronic constriction injury

This investigation was conducted as a pilot, proof-of-concept study aimed at establishing a reproducible CBD vapor exposure model and assessing feasibility of multi-tiered endpoint analysis (behavioral, histological, and molecular) within a single experimental framework. The group sizes were determined based on the exploratory nature of the study, ethical considerations governing animal use, and logistical constraints inherent to the custom vapor exposure system. The study was therefore optimized to detect consistent biological trends rather than to achieve inferential statistical power, and the findings should be interpreted accordingly.

### Animals

Adult, male Sprague–Dawley rats (Envigo, Indianapolis, IL), 150–175 g body weight on arrival were used for the sciatic nerve CCI model of neuropathic pain. The rats in this study were initially housed in pairs at a temperature of 22 ± 1 °C. The housing was provided in an accredited laboratory animal facility (LAF), where the rats had unrestricted access to food and water. Enrichment in the form of wooden blocks and red tubes were provided in cages. The LAF at SUNY/Buffalo are Association for Assessment and Accreditation of Laboratory Animal Care-accredited and meet the requirements established in the National Institutes of Health (NIH) Guide. A 12-h light/dark cycle was maintained, with the lights on from 06:00 to 18:00. Before baseline testing began, the rats were allowed a minimum of three days to acclimate to the animal facilities. The Institutional Animal Care and Use Committee of the University at Buffalo approved the experimental procedures followed. The NIH and the Committee for Research and Ethical Issues of IASP established guidelines for the moral treatment of animals, which were followed in this study (Zimmermann 1983). Every attempt was made to ensure adequate statistical power for the study while minimizing the number of rats used and minimizing pain and suffering. Humane endpoints were predefined in the approved IACUC protocol, consistent with the NIH Guide for the Care and Use of Laboratory Animals (8th Edition), but no animals met these criteria during the study.

### Vaping apparatus

**Fig. 1:**
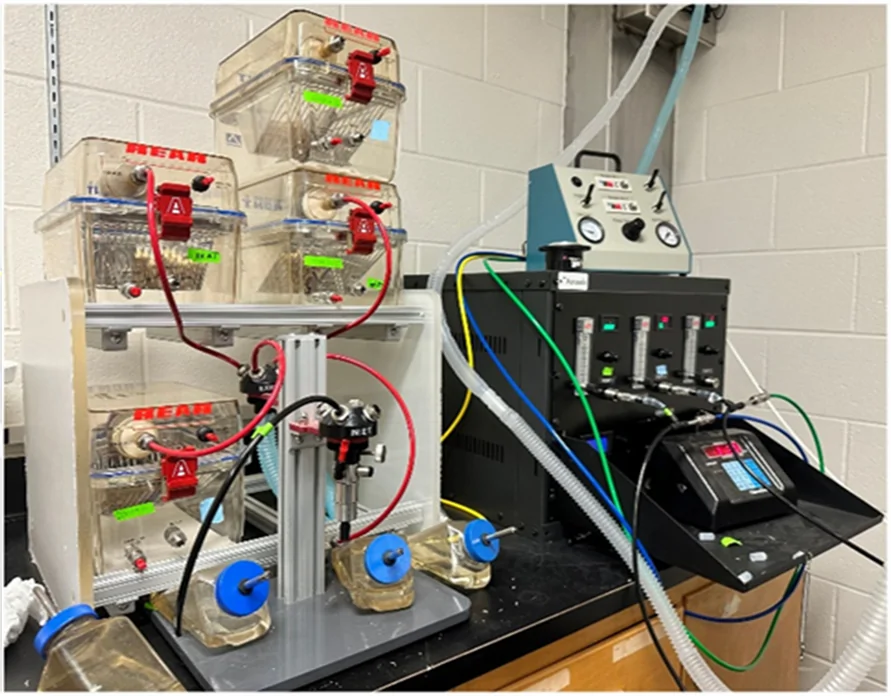
Automated vaping exposure system. A prototype of an automated exposure system (CSM-eSTEP) that includes an e-cigarette puffing machine, developed for small-scale inhalation exposure. In this study, we used the CH Technologies Modular BCU Exposure Chamber that are ideal for small-scale whole-body exposure studies. We adopted the Electronic Cigarette Aerosol Generator (ECAG) platforms that are designed specifically for electronic cigarettes and all vape products from the ground up. Here, instead of vaping liquids, e-cigarette cartridges were filled with CBD solution. This novel positive pressure system allows for more accurate physicochemical characterization and delivery at the exhaust port, reducing aerosol aging, and tube deposition, and eliminating artifacts that could be introduced from background air. The ECAG was designed to accommodate one-piece disposable electronic cigarettes (i.e., cig-alikes), in addition to the refillable tank/clearomizer 2nd thru 4th generation devices. CBD aerosols were generated from electronic cigarette (E-Cig) cartridges filled with 1 ml of CBD solution instead of vape fluid. CH Technologies aerosol generator generated CBD puffs (10 puffs for every 5 min, which repeated every 25 min up to 2 h)

The Automated Vaping Exposure System: For small-scale inhalation exposure applications, a prototype of an automated exposure system (CSM-eSTEP) was developed (Fig. 1) that includes an e-cigarette puffing machine. The vaping system can be set for any defined specific time duration, puff intervals, and puff volume equivalent to human vaping exposure (Farsalinos et al. 2013; Cunningham et al. 2016; Talih et al. 2015; Robinson et al. 2015; Dautzenberg & Bricard 2015). The setup permits in vivo vapor exposures by attaching a small animal cage housing rodents. With this specialized vaping device, the puff duration, puff volume, and puff length can be controlled using the push button display. It is significant to remember that there are well-established distinctions between the patterns of cigarette smoking and e-cigarette vaping among users (Ingebrethsen et al. 2012; Pouchez et al. 2018; Han et al. 2016). The e-cigarette, with a 0.5 Ω coil atomizer that operates at 3.2 V, produces aerosols with a high total particulate matter (TPM) concentration (measured in mass per puff) of up to 0.27 mg per 55 mL puff volume (Noël et al. 2018). The parameters of the e-cigarette device were examined, and the exposure chamber's conditions were standardized. For the in vivo experimental paradigm, the topography profile for a 30-min exposure was standardized at 55 mL puff volume, 3 s puff duration, and 30 s intervals where the e-liquid tested included the carrier solvents (i.e., PG and VG at a 50/50 ratio) alone, and in combination with 25 mg/mL CBD. This exposure regimen simulates the daily puff consumption and behavioral pattern of regular e-cigarette users, as previously reported (Cunningham et al. 2016).

### Chronic constriction injury of sciatic nerve

Rats were anesthetized with ketamine (75 mg/kg) and xylazine (10 mg/kg), i.p., prior to aseptic surgery with ⅓ bolus as maintenance dose when required. Chronic constriction injury (CCI) of the sciatic nerve was performed as described (Bennett and Xie 1988; Gerard et al. 2015; Ignatowski et al. 1999). The right hind leg of the rat was shaved. The shaved area was sterilized with consecutive applications of soap solution, 70% ethanol, and iodine solution, and lubricating ophthalmic ointment was applied to the eyes. With the rat lying on its chest/thorax, the right hind leg was extended and held in position with the femur at 90° to the spine. An incision was made in the skin parallel, but 3–4 mm below the femur, and the skin was freed from the muscle surrounding the incision by cutting through the connective tissue. The connective tissue between the gluteus superficialis and the biceps femoris muscles was separated by blunt dissection, exposing the common sciatic nerve at the level of the middle of the thigh. A retractor was used to widen the gap between the two muscles, allowing clear visualization of the sciatic nerve. Proximal to the sciatic nerve's trifurcation, about 7 mm of the nerve was freed of adhering tissue using curved blunt-tipped forceps, and 4 ligatures (4.0 chromic gut) were tied loosely around the common portion of the nerve with about 1 mm spacing. The length of the nerve thus affected was 4–5 mm long. Great care was taken to tie the ligatures such that the diameter of the nerve was seen to be just barely constricted when viewed with 40X magnification. The incision was closed in layers by using sutures (3.0 chromic gut) to close the muscle layer and staples to fasten the skin. Finally, an iodine solution was used to sterilize the wound. The sham rats received surgery, whereby the sciatic nerve was exposed and freed of adherent tissue/muscle, however, no ligatures were placed. All surgeries were performed between 09:00–12:00. The rats were observed closely during the anesthesia recovery period. They were recovered in separate heated cages with absorbent blue-pads covering the standard rat husbandry bedding to prevent the unconscious rats from choking upon waking. Wet chow in dishes was placed in the cages for easy access after surgery.

### Randomization, blinding, and analgesia

Animals were randomly assigned to treatment groups (Sham + Air, CCI + Air, CCI + PGVG, and CCI + CBD vapor) using a random number generator before vapor exposure. Behavioral testing and histopathological analyses were performed by experimenters blinded to treatment allocation to minimize bias.

Surgeries were performed under ketamine/xylazine anesthesia, and no postoperative analgesics were administered because systemic analgesics (e.g., opioids, NSAIDs, or local anesthetics) can interfere with nociceptive sensitivity and confound pain-behavior outcomes in neuropathic pain models. This decision aligns with established CCI protocols (Bennett and Xie 1988; Zimmermann 1983), which omit postoperative analgesia to preserve the validity of pain-response assessments.

Post-surgical animals were monitored twice daily for 72 h and thereafter daily for signs of distress, infection, or autotomy; none met humane endpoint criteria or required intervention.

### Measurements of pain behaviors

Rats that had CCI exhibited abnormal hind paw posture (toes held together, plantar flexed, and paw everted), as well as frequent guarding, licking, and shaking of the injured hind paw, all of which point to the presence of spontaneous pain.

### Thermal hyperalgesia measurements

At specified times post-CCI, the thermal nociceptive threshold was measured in each hind paw. Thermal hyperalgesia (increased sensitivity to noxious sensory/thermal stimuli) was measured by determining changes in paw withdrawal latency (PWL) using a plantar algesia apparatus (model #33, Analgesia Meter, IITC Life Science Instruments, Woodland Hills, CA) (Hargreaves et al. 1988). PWL was measured using an intense heat source to stimulate thermal receptors in the sole of the foot. A maximal automatic cut-off latency of 15 s was used to prevent tissue damage. Rats were placed in Plexiglas chambers, on top of a temperature maintained (32 ± 0.1 °C) glass surface. Rats were acclimated to the testing apparatus for 7–10 min (or until exploratory behavior ceased), and measurements of the thermal withdrawal threshold were taken for each hind paw. Baseline latencies were determined before experimental treatment for all animals as the mean of three separate trials, taken 4-, 2-, or 1-day pre-surgery and/or the day of the surgery (prior to surgery, day 0). Paw withdrawal responses were measured on day-2 post-surgery to ensure development of peripheral hypersensitivity in the affected (ipsilateral) hind paw. Only rapid hind paw movements away from the thermal stimulus (with or without licking of hind paw) were considered to be a withdrawal response. Paw movements associated with weight shifting or locomotion were not counted. Each hind paw was measured three times at ≥ 4-min intervals, and the averaged values were used to compute thermal hyperalgesia. A “difference score” generated from subtracting the contralateral PWL from the ipsilateral PWL was used as the index of hyperalgesia. All measurements were recorded between 07:00 and 12:00 by the same female researcher for consistency.

### Mechanical allodynia/hyperalgesia measurements

Calibrated Semmes–Weinstein (von Frey) monofilaments (Stoelting Co., Wood Dale, IL) were used to measure the hind paw withdrawal threshold (in grams force) in rats (Gerard et al. 2015). Rats were placed in elevated Plexiglas chambers with wire-mesh bottoms and were acclimated for 7–10 min (or until exploratory behavior ceased). Calibrated filaments were applied to the plantar surface of the hind paws, and paw withdrawal reflexes were recorded for each hind paw. The criterion response was reflexive withdrawal without stepping. Two von Frey monofilaments (bending forces in mN(gram forces), 4.08(1) and 5.18(15)), were applied perpendicular to the central region of the plantar surface in ascending order (Leite-Almeida et al. 2009); filaments were depressed until they bent. Each hind paw was measured 10 times with brief probe application at ≥ 1 s intervals. Regular movement and inadvertent movements were not considered as a direct response to the filament application. The differential response between the baseline and post-surgery values for each hind paw (injured [ipsilateral] and control [contralateral]) was a measure of mechanical or tactile allodynia (1 g filament) and mechanical hyperalgesia (15 g filament). The individual paw withdrawal threshold was determined by 2 days of baseline testing performed between 07:00 and 12:00; these baseline values were averaged to provide one value. Paw withdrawal responses were measured at various days post-surgery for a 17-day period. The frequencies of withdrawals were expressed as percentages (number of paw withdrawals/number of trials X 100) (Moon et al. 1999). These measurements were carried out by the same female researcher for consistency. In order to reduce potential confounders such as rat location during testing, rats were alternated in chambers each testing period.

### Cold allodynia measurements

Cold allodynia was assessed using acetone as previously described (Griffiths et al. 2018). The testing apparatus employed for cold allodynia assessment remained consistent with that used for mechanical allodynia. Acetone (50 µl) was applied to the plantar surface of each hind paw using a P200 pipette tip attached to a 5 cc syringe, and a stopwatch started.

Responses were categorized based on the following criteria:

- 0 = no response
- 1 = single withdrawal (lifting, flicking, or stamping the paw)
- 2 = multiple or prolonged withdrawals
- 3 = paw withdrawal and licking on the plantar surface

The duration of responses was evaluated for 40 s following acetone application, starting with the right hind paw of each rat, followed by the left hind paw, and this process was repeated three times. A minimum interval of 4 min was observed before subsequent acetone applications on the same hind paw. The individual paw withdrawal response was determined by 2 days of baseline testing performed between 07:00 and 12:00; these baseline values were averaged to provide one value. Paw withdrawal responses were assessed on various days post-surgery over a 17-day period. These assessments were consistently conducted by the same female researcher. Rat chamber occupation during testing was alternated each testing period to minimize potential confounding factors.

### Serum collection

Euthanasia by decapitation in absence of anesthesia was immediately followed by trunk blood collection into sterile 50 ml tubes that were kept on ice for 15–30 min. The tubes of blood were then centrifuged at 4 °C at 3000 rpm for 10 min. The clear serum was subsequently aliquoted into 2 sterile tubes and stored at –80 °C for further analysis. Serum samples were cataloged by numbers for blinding of samples in subsequent assays.

### Bronchoalveolar lavage (BAL) fluid

Bronchoalveolar lavage (BAL) fluid (1–2 mL) was collected using an 18-G catheter needle attached to a 5 mL syringe; the catheter was inserted into the trachea and three washes with saline (1X PBS) was performed. The lavage fluid collected into a sterile 15 ml tube on ice was centrifuged at 400 × g at 4 °C for 7 min. Following centrifugation, the supernatant was collected and stored at –80 °C for further analysis. BAL samples were cataloged by numbers for blinding of samples in subsequent assays.

### Lung tissue

Upon completion of the BAL collection, the lower right lobe of the rat (on the left when viewed ventrally) was collected, snap-frozen in liquid nitrogen, and stored at –80 °C for gene expression studies using QPCR. Next, a catheter was inserted into the trachea, and the lungs were infused with 10% neutral buffered formalin (NBF) for fixation. Following formalin infusion, the trachea was clamped, and the lungs, heart, and trachea were removed from the body cavity and submerged in a tube containing 10% NBF, stored at 4 °C for 6 days, after which the dehydration process for paraffin embedding was initiated for histology. Hematoxylin and eosin-stained lung sections were scanned at 40X resolution using an Aperio ScanScope CS System. Captured and exported images were displayed at different effective magnifications during image processing: Broad, full-field views showing large alveolar regions and small bronchioles correspond to 10 × magnification; the intermediate views that show alveolar septal thickening correspond to 20 × magnification; and the close-up fields highlighting RBC extravasation and alveolar macrophages represent 40 × magnification. Scale bars corresponding to these effective magnifications are included in the images. Histopathological evaluation was conducted descriptively by independent observers to ensure consistent interpretation. The intent of this analysis was to provide qualitative confirmation of pulmonary injury patterns consistent with the observed inflammatory and molecular changes, rather than to derive quantitative pathology scores.

### RNA extraction

RNA was extracted from lung tissue samples using the TRIzol® reagent. 1 mL of TRIzol® reagent was added to lung tissue (~ 10 mg weight), and tissues were homogenized using Benchmark D1030 BeadBug 3-Position Microtube Homogenizer for 60 s at 4000 rpm, followed by standard extraction procedure as per the Manufacturer’s protocol. The RNA pellet was reconstituted with 25 µL of DEPC H_2_O and dissolved by vortexing. The amount of RNA was quantified using a NanoDrop ND-1000 spectrophotometer (Nano-Drop™, Wilmington, DE), and isolated RNA was stored at −80 °C until it was used.

### Real-time quantitative (RT-q) PCR

500 ng of the total RNA that was extracted as described above was utilized for the All-in-One Universal RT Master Mix synthesis kit (Lamba Biotech) following the Manufacturer’s protocol. One microliter of the resultant cDNA from the RT reaction was employed as the template in PCR reactions using well-validated PCR primers obtained from IDT-Integrated DNA Technologies. We used the SYBR® Green master mix that contained dNTPs, MgCl_2_, and DNA polymerase (Bio-Rad, Hercules, CA). The final primer concentration used in the PCR was 0.1 µM. The following were the PCR conditions: 95 °C for 3 min, followed by 40 cycles of 95 °C for 40 s, 60 °C for 30 s, and 72 °C for 1 min; the final extension was at 72 °C for 5 min. Gene expression was calculated using the comparative CT method. To account for variations in RNA input quantity, measurements were performed on an endogenous reference gene, β-actin. The threshold cycle (Ct) of each sample was determined, the relative level of a transcript (2ΔCt) was calculated by obtaining ΔCt (test Ct − β-actin Ct), and transcript accumulation index (TAI) was calculated as TAI = 2^−ΔΔCT^ (Livak and Schmittgen 2001).

### ELISA

We quantitated levels of TNF-α, IL-6, and Phospho ERK-1 in serum and BAL samples obtained from all animal groups using the following commercially available ELISA kits following detailed manufacturer’s instructions: TNF-α (Rat TNF-alpha DuoSet ELISA; Catalog #DY510; R & D Systems Inc, Minneapolis, MN), IL-6 (Rat IL-6 ELISA Kit – Quantikine; Catalog #R6000B, R & D Systems Inc, Minneapolis, MN), and Phospho ERK-1 (Human/Mouse/Rat Phospho-ERK1 (T202/Y204) DuoSet IC ELISA; Catalog #: DYC1825-2).

### Nitric oxide

Endogenous NO production was measured in serum and BAL samples obtained from all animal groups using the commercially available Griess Assay (Promega Inc, Madison, Wisconsin; Catalog #G2930). The Griess assay provides a reliable and quantitative estimate of the nitric oxide output. Serum nitrite levels are measured in 50 µl of serum and BAL samples aliquoted in 96-well microplate followed by addition of 50 μl Griess reagent. Total nitrite is estimated from a standard absorbance curve at 592 nm using a spectrophotometer, and NO_2_ concentration was determined using NaNO_2_ standards. The values reported are the values detected in the directly harvested BAL fluids and sera, without correction for dilution.

### Statistical analysis

Statistical analysis for the behavioral data in this study was performed using InVivoStat (v4.10) software, a freely available statistical software package that uses R as its statistics engine and is designed specifically for analyzing data generated from animal experiments. Comparisons between two groups used extended paired t-tests, while multiple group comparisons utilized 1-way ANOVA, 2-way ANOVA, or 2-way repeated measures ANOVA, all using a mixed model approach, followed by Planned Comparisons with LSD post-hoc testing. The QPCR and ELISA data analysis was done using Prism (GraphPad Ver 9.0). All QPCR and ELISA experiments were done in triplicate, and data are presented as mean ± standard error of the mean (SEM). A p value of ≤ 0.05 was considered to be statistically significant.

## Results

### Thermal hyperalgesia

**Fig. 2:**
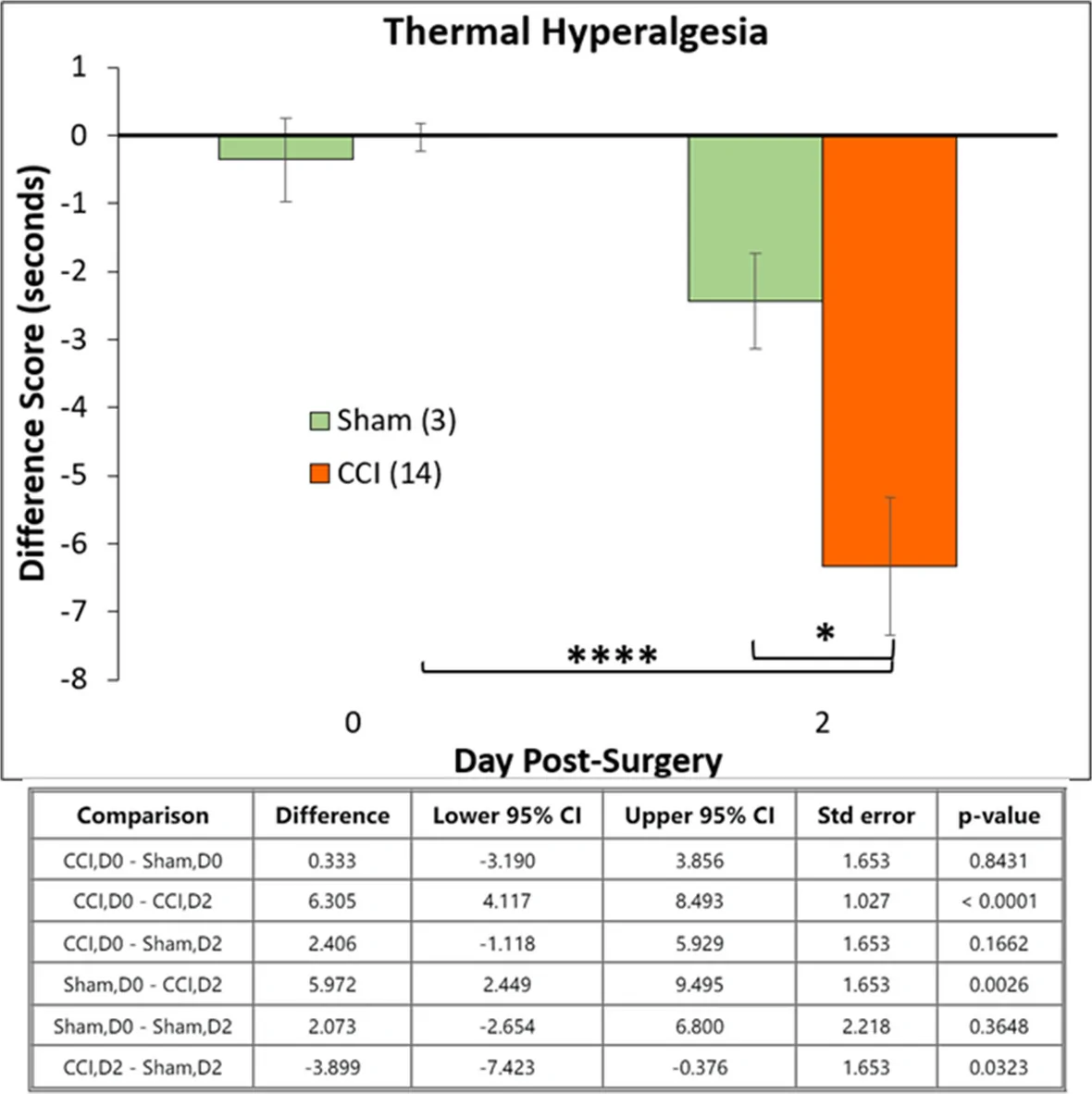
Thermal Hyperalgesia – CCI vs Sham. Assessment of thermal hyperalgesia two days after sciatic nerve CCI. Data are presented as the difference score of ipsilateral/experimental–contralateral/control hind paw withdrawal latency in seconds. Each bar is expressed as the mean ± S.E.M. (number of rats in parentheses). Statistical significance different from sham-operated rats was reached at *p = 0.0323, and difference from respective baseline (day 0) was reached at ****p < 0.0001 using a 2-way Repeated Measures Mixed Model approach, with Surgery as the treatment factor and Day as the repeated factor. This was followed by planned comparisons on the predicted means to compare the levels of the effect Surgery*Day; the Table shows all pairwise comparisons. CCI, chronic constriction injury

All the rats underwent surgery on the right hind paw on day 0; three rats had a sham surgery performed and the remaining 14 rats had CCI surgery. Peripheral hypersensitivity to a noxious light stimulus, or thermal hyperalgesia, developed in the ipsilateral (operated) hind paw of CCI rats. The results are expressed as a difference score (ipsilateral-contralateral paw withdrawal score in sec to a noxious stimulus), with a negative difference score indicating peripheral hypersensitivity (Fig. 2). Baseline latencies were determined before CCI surgery for all animals as the means of three separate trials, taken one, four-days pre-surgery and on the day of surgery (days −4, −1, and 0).

The data analysis for this experiment employed a 2-way Repeated Measures Mixed Model approach. In this analysis, Surgery was designated as the treatment factor, while Day served as the repeated factor. Following this, planned comparisons were conducted on the predicted means to assess the levels of the effect Surgery*Day. In summary, there exists a statistically significant overall difference between the levels of Day and Surgery at the 5% significance level. Planned comparisons determined statistical significance at *p < 0.05 and ****p < 0.0001, as shown in Fig. 2.

All pairwise comparisons were conducted without adjustment for multiplicity shown in the Table below Fig. 2. Statistically significant differences were observed at the 5% significance level in the following pairwise tests: CCI, Day 0—CCI, Day 2; Sham, Day 0—CCI, Day 2; and CCI, Day 2—Sham, Day 2. However, only planned comparisons were considered (i.e., CCI, Day 0 vs CCI, Day 2; and CCI, Day 2 vs Sham, Day 2).

### Mechanical allodynia and hyperalgesia

**Fig. 3:**
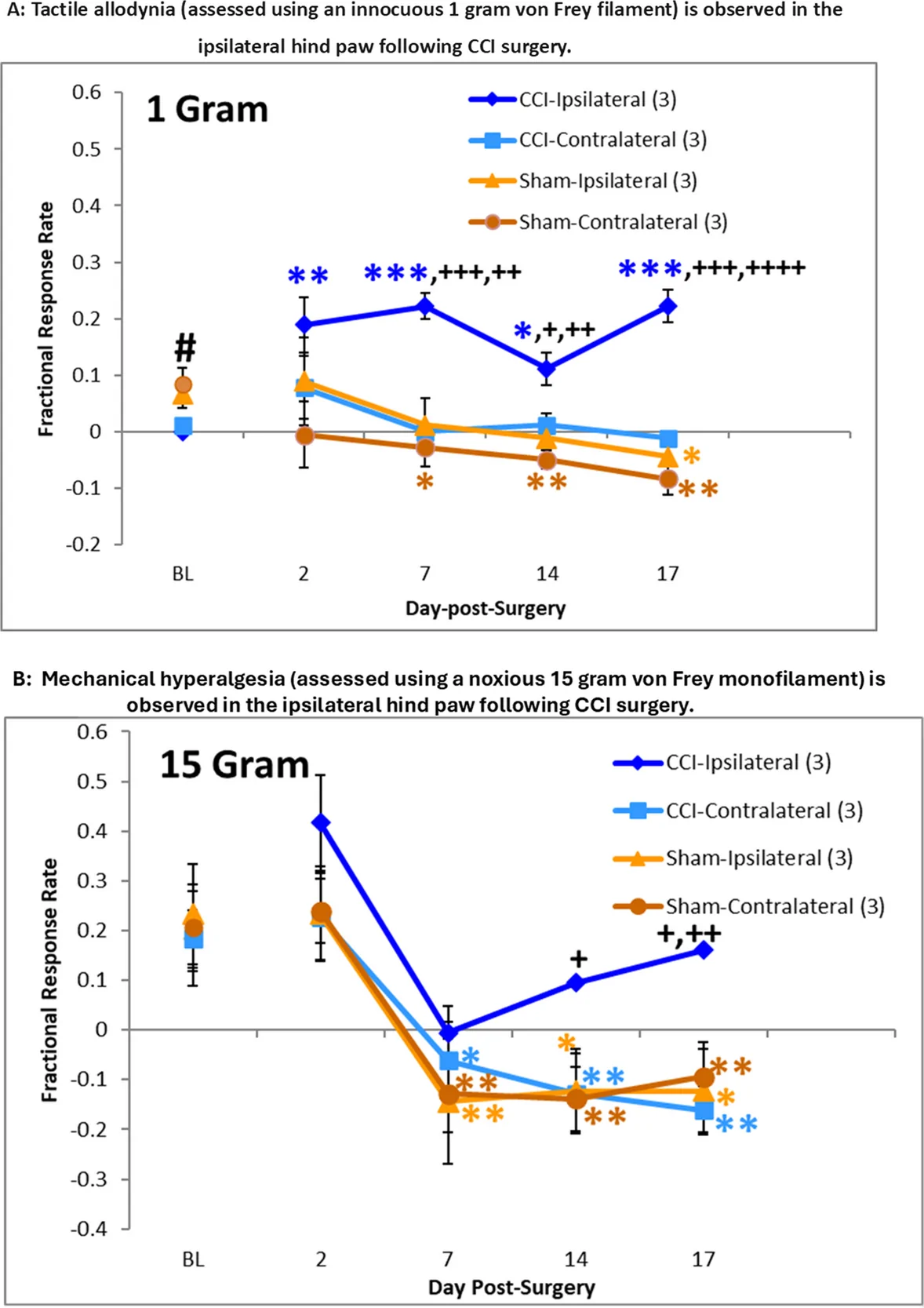
Assessment of mechanical stimulation. **A** Tactile allodynia (assessed using an innocuous 1 g von Frey filament) is observed in the ipsilateral hind paw following CCI surgery. CCI rats developed ipsilateral hind paw tactile allodynia at days 7, 14, and 17 after ligature placement. Initial 2-way Repeated Measures Mixed Model analysis determined a Surgery effect, F(1, 4) = 10.33, *P* = 0.0325, and a Surgery*Day effect, F(4, 46) = 3.37, *P* = 0.0169. Planned comparisons on the predicted means determined significance for CCI-ipsilateral hind paw responses at levels of *p = 0.0461, **p = 0.002, ***p = 0.0005 when comparing to respective baseline (BL) values. Sham-ipsilateral at day 17 as compared to respective BL, **p = 0.0014; Sham-contralateral as compared to respective BL at day 7, *p = 0.0204; day 14, **p = 0.0071; and day 17, **p = 0.0014. Employing a 2-way ANOVA approach at each day followed by LSD post-hoc test and analysis of planned comparisons determined: Sham-contralateral exhibited significance at #*p* = 0.0036 compared to respective CCI-contralateral BL; Day 2 yielded no significant differences; Day 7 demonstrated significance at +++*p* ≤ 0.001 vs CCI-contralateral (*p* = 0.001) and vs Sham-contralateral (*p* = 0.0005), and ++*p* < 0.01 vs Sham-ipsilateral (*p* = 0.0014); Day 14 revealed significance at +*p* < 0.05 vs CCI-contralateral (*p* = 0.0358) and vs Sham-ipsilateral (*p* = 0.0151), and ++*p* < 0.01 vs Sham-contralateral (*p* = 0.0036); Day 17 displayed significance at +++*p* < 0.001 vs CCI-contralateral (*p* = 0.0002), and ++++*p* < 0.0001 vs Sham-ipsilateral and vs Sham-contralateral. **B** Mechanical hyperalgesia (assessed using a noxious 15 g von Frey monofilament) is observed in the ipsilateral hind paw following CCI surgery. Two-way repeated measures mixed model approach determined a Day effect, F(4, 46) = 17.71, *P* < 0.0001. Planned comparisons revealed significance for Sham-ipsilateral hind paw responses at the levels of *p < 0.05, **p < 0.01 compared to respective baseline (BL) values: Day 7, *p* = 0.0071; Day 14, *p* = 0.0104; Day 17, *p* = 0.0104. Sham-contralateral as compared to respective BL at day 7, **p = 0.0042; day 14, **p = 0.0033; and day 17, **p = 0.0084. CCI-contralateral as compared to respective BL at day 7, *p = 0.0263; day 14, **p = 0.0067; and day 17, **p = 0.0033. The data at each day underwent analysis utilizing a 2-way ANOVA approach with Surgery and Hind paw as the treatment factors. Subsequent planned comparisons of the predicted means were conducted to compare the levels of the Surgery*Hind paw interaction: Baseline (BL) values, NS; Day 2, NS; and Day 7, NS. Day 14, +*p* < 0.05 CCI-ipsilateral vs Sham-contralateral (*p* = 0.0425); CCI-ipsilateral trended toward significance vs Sham-ipsilateral, *p* = 0.0555 and vs CCI-contralateral, *p* = 0.0508. Day 17, ++*p* = 0.0082 CCI-ipsilateral vs CCI-contralateral, and +*p* < 0.05 CCI-ipsilateral vs Sham-ipsilateral (*p* = 0.0154) and Sham-contralateral (*p* = 0.0244)

Following CCI surgery, mechanical allodynia, as assessed using an innocuous 1 g von Frey filament, was observed in the ipsilateral hind paw. Utilizing a 2-way repeated measures mixed model approach, a statistically significant overall difference between the levels of Surgery and Surgery*Day was determined. Planned comparisons revealed significance levels of *p < 0.05, **p < 0.01, ***p < 0.001 when comparing to respective baseline (BL) values (Fig. 3A). Employing a 2-way ANOVA approach at each day followed by LSD post-hoc test and analysis of planned comparisons determined: Shams exhibited significance at *p < 0.05 compared to respective CCI baselines; Day 2 yielded no significant differences; Day 7 demonstrated significance at +++*p* < 0.001 vs CCI-contralateral and vs Sham-contralateral, and ++*p* < 0.01 vs Sham-ipsilateral; Day 14 revealed significance at +*p* < 0.05 vs CCI-contralateral and vs Sham-ipsilateral, and ++*p* < 0.01 vs Sham-contralateral; and Day 17 displayed significance at +++*p* < 0.001 vs CCI-contralateral, ++++*p* < 0.0001 vs Sham-ipsilateral, and vs Sham-contralateral (Fig. 3A).

After CCI surgery, mechanical hyperalgesia was observed in the ipsilateral hind paw, assessed using a ‘noxious’ 15 g von Frey monofilament. Statistical analysis employing a 2-way repeated measures mixed model approach determined a statistically significant overall difference between the levels of Day. Planned comparisons revealed significance levels of *p < 0.05, **p < 0.01 compared to respective BL values (Fig. 3B). The data at each day underwent analysis utilizing a 2-way ANOVA approach with Surgery and Hind paw as the treatment factors. Subsequent planned comparisons of the predicted means were conducted to compare the levels of the Surgery*Hind paw interaction: BL values exhibited no significant differences; Day 2 showed no significant differences; and Day 7 demonstrated no significant differences. On Day 14, significance was observed at +*p* < 0.05 comparing CCI-ipsilateral to CCI-contralateral, Sham-ipsilateral, and Sham-contralateral. On Day 17, significance was observed at ++*p* < 0.01 comparing CCI-ipsilateral to CCI-contralateral, and at +*p* < 0.05 comparing to Sham-ipsilateral and Sham-contralateral (Fig. 3B).

**Fig. 4:**
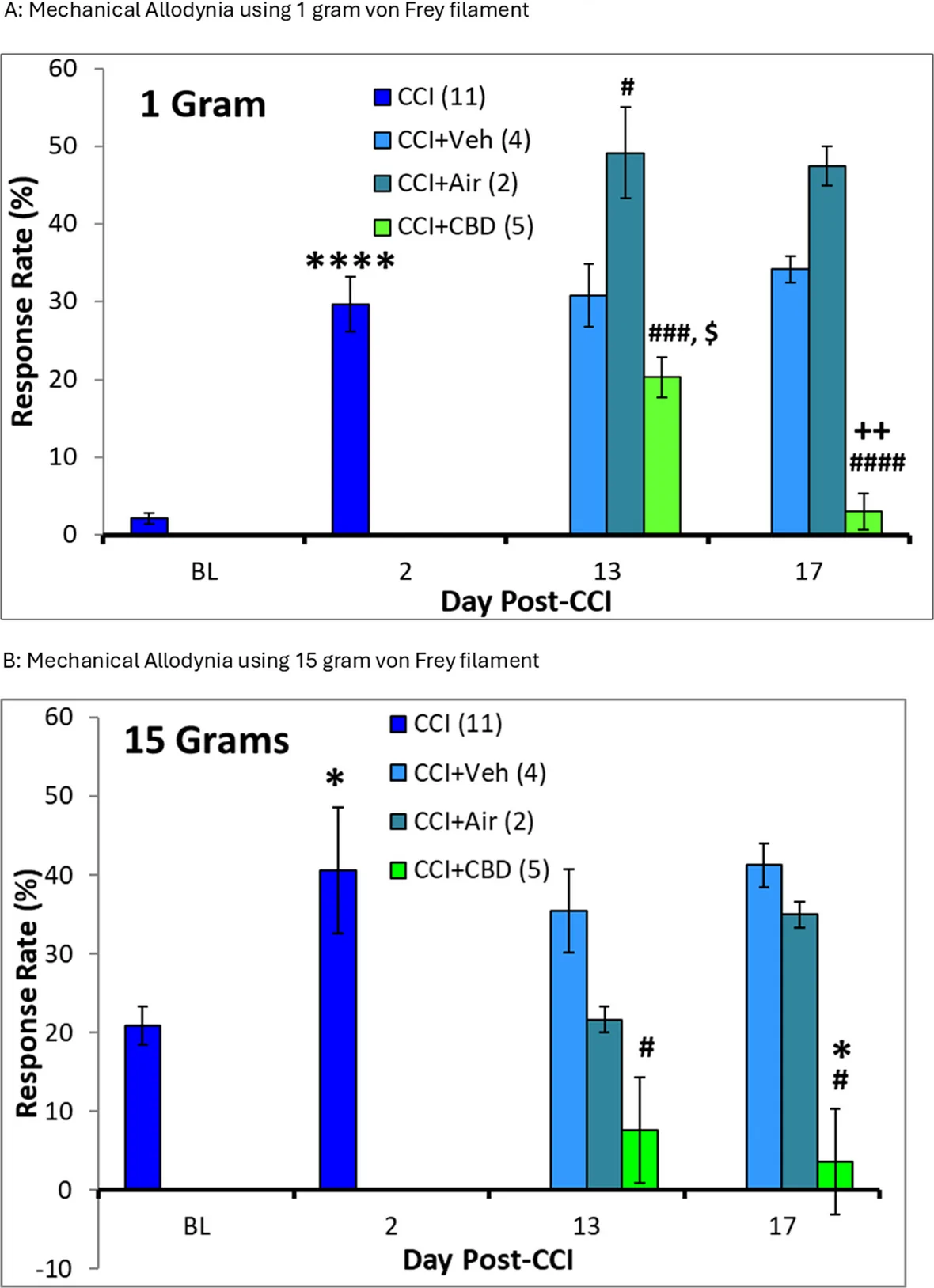
CBD vapor alleviates CCI-induced mechanical allodynia and hyperalgesia in the ipsilateral hind paw. **A** InVivoStat analysis of Mechanical Allodynia using 1 g von Frey filament: BL vs D2: Extended paired t-test (= repeated measures mixed model approach): ****p < 0.0001. D2-D17: 2-way Mixed Model approach used with Treatment as the treatment factor and Day as the repeated factor. This was followed by Planned Comparisons on the predicted means to compare the levels of the effect Treatment Day: D13: ###*p* = 0.0008, CCI-CBD vs CCI-Air; $*p* = 0.0805, CCI-CBD vs CCI-Veh; #*p* = 0.0226, CCI-Veh vs CCI-Air. D17: ####*p* < 0.0001, CCI-CBD vs CCI-Veh and CCI-Air. ++*p* = 0.0068, CCI-CBD-D13 vs CCI-CBD-D17. **B** InVivoStat analysis of Mechanical Allodynia using 15 g von Frey filament: BL vs D2: Extended paired t-test (= repeated measures mixed model approach): *p = 0.0353. D2-D17: 2-way Mixed Model approach used with Treatment as the treatment factor and Day as the repeated factor. This was followed by Planned Comparisons on the predicted means to compare the levels of the effect Treatment Day: D13: #*p* = 0.0502, CCI-CBD vs CCI-Veh. D17: *p = 0.0112, CCI-CBD vs CCI-Veh and #*p* = 0.0733, CCI-CBD vs CCI-Air

The analysis of mechanical sensitivity involved the use of both an innocuous 1 g von Frey filament and a noxious 15 g von Frey monofilament. Initially, a comparison between BL and Day 2 (D2) was conducted using an extended paired t-test, revealing a significant difference (****p < 0.0001, 1-g; *p < 0.05, 15-g) for both filaments (Fig. 4A&B). Subsequently, a 2-way Mixed Model approach was applied for the period between Day 2 and Day 17 (D2-D17), with Treatment as the treatment factor and Day as the repeated factor. Planned Comparisons on the predicted means unveiled significant differences on Day 13 (###*p* < 0.001) for the 1-g filament, where CCI + CBD rats responded much less to filament probing compared to CCI + Air rats, with a similar trend observed ($*p* = 0.0805) between CCI + CBD and CCI + Veh rats (Fig. 4A). Moreover, on Day 17 (####*p* < 0.0001), significant difference was noted for both filaments, with CCI + CBD differing from CCI + Veh and CCI + Air. Additionally, comparison between CCI + CBD-D13 and CCI + CBD-D17 indicated significance at the ++*p* < 0.01 level for the 1-g filament (Fig. 4A), indicating continued reduction of allodynia.

Similarly, for the noxious 15-g von Frey monofilament, the analysis revealed significance (*p < 0.05, *p* = 0.0353) between BL and Day 2 (D2) through an extended paired t-test. For the period between Day 2 and Day 17 (D2-D17), a 2-way Mixed Model approach was utilized, with Treatment as the treatment factor and Day as the repeated factor. Planned Comparisons on the predicted means showed significance on Day 13 (#*p* < 0.05), where CCI + CBD differed from CCI + Veh. Furthermore, on Day 17, a significant difference was observed (#*p* < 0.05), with the CCI + CBD group showing reduced responsiveness to a noxious stimulus as compared to both CCI + Veh and CCI + Air groups (Fig. 4B). Additionally, comparison between CCI + CBD-D13 and CCI + CBD-D17 indicated no significant difference for the noxious 15-g von Frey monofilament (Fig. 4B).

### Cold allodynia

**Fig. 5:**
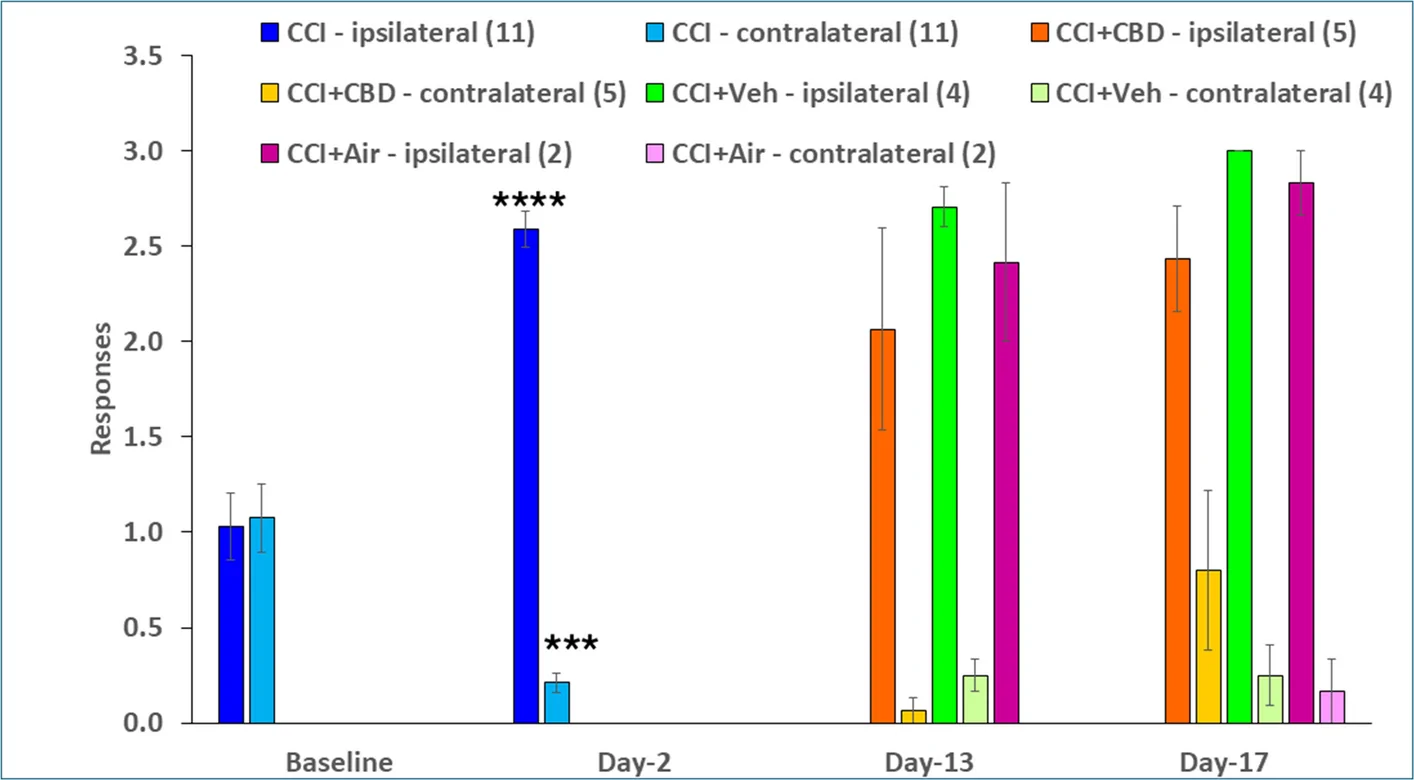
Cold Allodynia. A comparison between baseline (BL) and Day-2 ipsilateral measurements utilized an extended paired t-test, employing a repeated measures mixed model approach, revealing a significant increase in cold sensitivity after CCI surgery (****p < 0.0001). Contralateral measurements showed decreased cold sensitivity on Day-2 compared to baseline (***p = 0.0008). Repeated measures 2-way ANOVA for ipsilateral and contralateral hind paws on Days 2–17 showed no significant changes

The comparison between BL and Day-2 ipsilateral measurements utilizing an extended paired t-test, employing a repeated measures mixed model approach, revealed a significant difference with ****p < 0.0001, demonstrating increased cold sensitivity after CCI (Fig. 5). The comparison between BL and Day-2 contralateral measurements, utilizing the same statistical methodology, yielded a significant result with ***p < 0.001, showing decreased contralateral cold sensitivity after CCI. Subsequent analysis using repeated measures 2-way ANOVA for ipsilateral hind paws on Days 2–17 and contralateral hind paws on Days 2–17 showed non-significant findings for ipsilateral measurements (Fig. 5). Consequently, it was observed that CBD vapor did not exhibit efficacy in alleviating cold allodynia.

### Lung histopathology

**Fig. 6:**
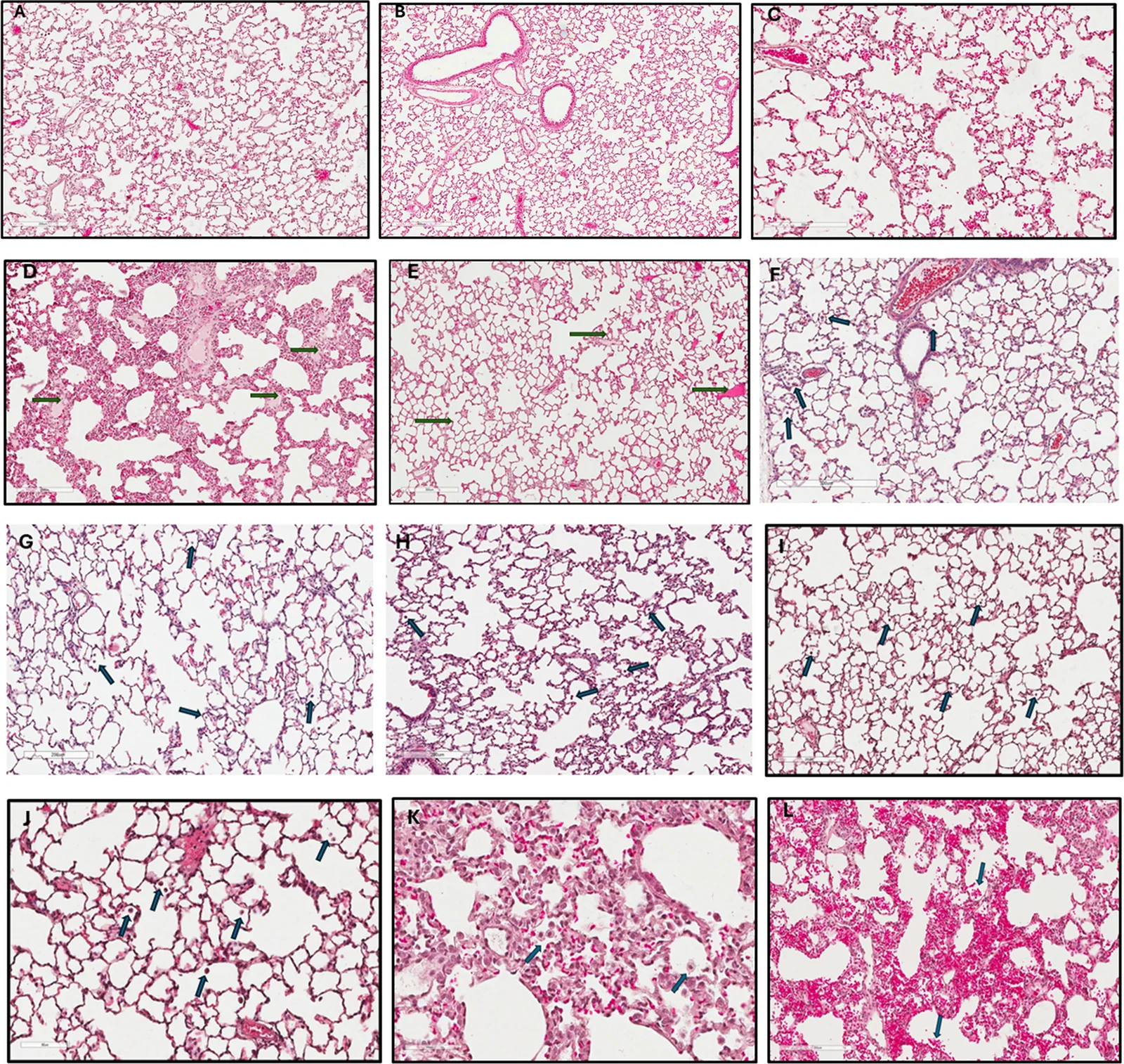
Lung histology via H&E staining from Sham and CCI rats. **A** & **B** Sham-operated rats exhibited histologically normal lung structure, characterized by intact bronchioles, arteries, capillaries, alveoli, and veins. Magnification: 10x; Scale bars in (**A**) = 200 µm and (**B**) = 300 µm. **C**-**E** CCI rats revealed thickening of the alveolar walls, RBCs in alveoli, and hyaline material (green arrows). Magnification: 20x; Scale bars in (**C** + **D**) = 200 µm and (**E**) = 300 µm. **F**–**H** H&E staining of lung tissue from CCI rats treated with PGVG vapor vehicle for two weeks. Lung tissue showed enlarged alveoli, presence of alveolar macrophages (green arrows), and (**H**) thickened alveolar walls. Magnification: 20x; Scale bars in (**F**) = 300 µm and (**G** + **H**) = 200 µm. **I**-**L** H&E staining of lung tissue from CCI rats treated with CBD vapor for two weeks. Lung tissue showed (**I**-**L**) enlarged alveoli, **J**-**L** thickened alveolar walls, **I**-**K** presence of alveolar macrophages (green arrows), and (**L**) RBCs in alveoli (arrows). Magnification in (**I**) = 20x, in (**J**-**L**) = 40x; Scale bars in (**I** + **L**) = 200 µm, **J** = 80 µm, and **K** = 60 µm

The left lower lobes of the lungs from all rats were collected for histological H&E staining. Sections 10 microns thick were cut, stained, and observed under a light microscope at various magnifications for histopathological analysis. Sham-operated rats predominantly displayed normal morphology without significant abnormalities (Fig. 6A&B).

Conversely, control CCI rats exhibited RBC leakage into alveoli, potentially attributed to trauma/stress from the constriction injury. Additionally, protein leakage and the presence of hyaline material were observed in select alveoli across all rats, possibly resulting from capillary trauma (Fig. 6C-E).

Lungs from rats in the vehicle control group (CCI + PGVG) appear similar to the CCI group, with the following exceptions: the presence of alveolar macrophages and the lack of RBCs in alveoli (Fig. 6F-H).

In the experimental group comprising CCI rats treated with CBD vapor for two weeks, observations revealed thickened alveolar walls in the central region, alongside instances of ruptured alveoli or disruptions in the alveolar-capillary barrier, resulting in enlarged alveoli. Notably, some alveoli contained RBCs and/or alveolar macrophages, with the presence of hyaline material (Fig. 6I-L).

Although formal histopathological scoring was not performed, the characteristic features of alveolar septal thickening, peribronchiolar infiltration, and epithelial disorganization were consistently observed across all CBD-exposed animals, providing semi-quantitative reproducibility of the pathological phenotype. These morphological findings align with the elevated expression of inflammatory mediators and NLRP3 pathway activation identified in molecular analyses.

### Cytokine levels in BAL vs serum and in lung tissue

**Fig. 7:**
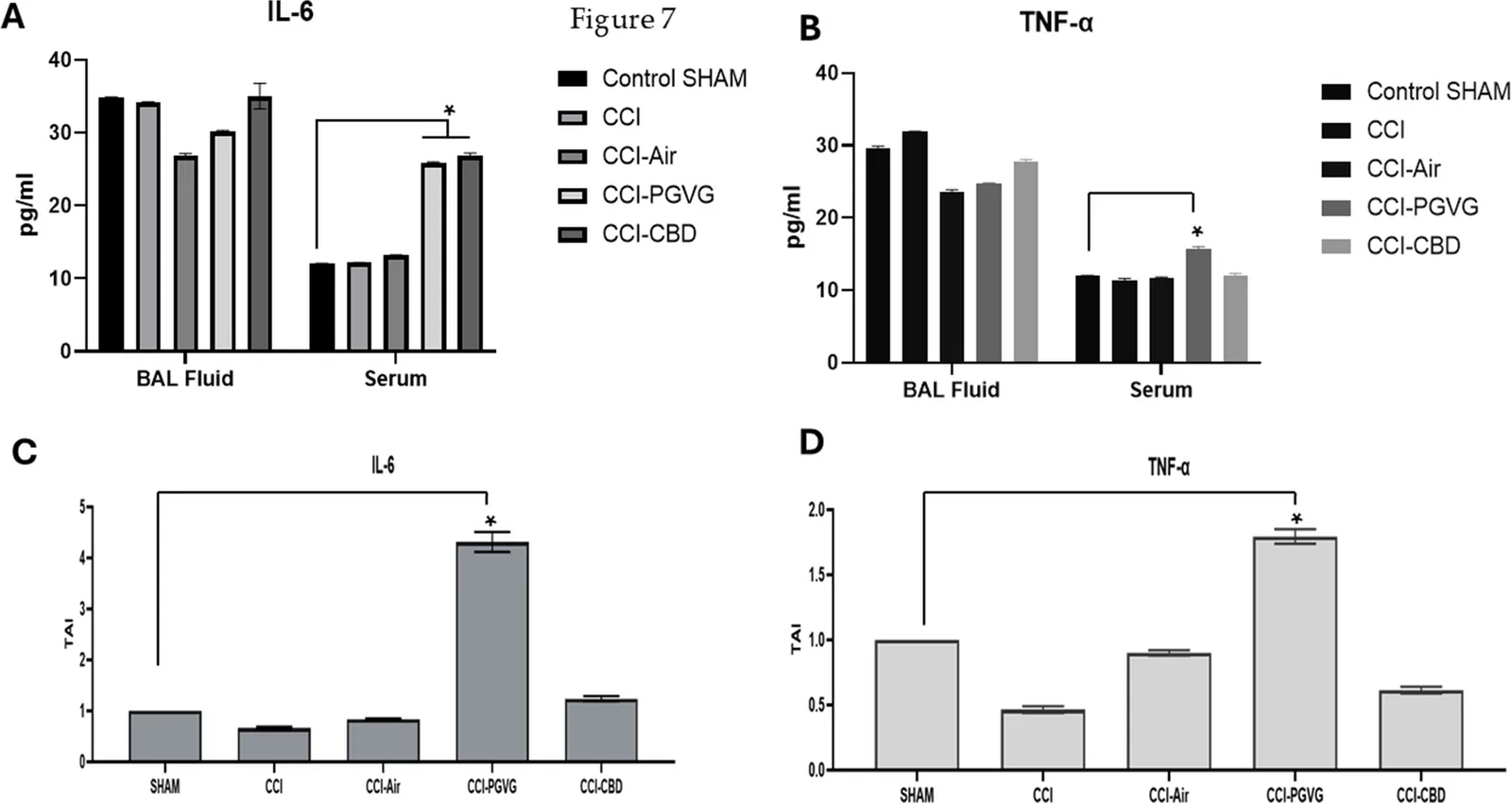
Levels of proinflammatory cytokines. Protein levels of IL-6 (**A**) and TNF-α (**B**) in BAL fluid and sera from SHAM and CCI rat groups. Bars represent mean ± S.E.M. values in pg/ml as per ELISA kit analysis. RT-QPCR analysis of mRNA for IL-6 (**C**) and TNF-α (**D**) in BAL fluid and sera from SHAM and CCI rat groups. Bars represent mean ± S.E.M. Transcript accumulation index, TAI values

We obtained lung BAL fluid from rats in the experimental groups namely, SHAM, CCI, CCI + Air, CCI + PGVG and CCI + CBD. The levels of pro-inflammatory cytokines, TNF-α, IL-6, and Nitric Oxide (NO), were measured in serum and BAL fluid from the CCI groups and compared with levels in the SHAM controls. We observed that the levels of IL-6 [31.512 ± 1.14 pg/ml (BAL) vs 17.82 ± 2.907 pg/ml (serum) *p* < 0.002)] and TNF-α [27.45 ± 1.22 pg/ml (BAL) vs 12.47 ± 0.62 pg/ml (serum) *p* < 0.0001)] were significantly higher in BAL fluid as compared to the levels of these cytokines in serum (Fig. 7A & B). We observed no significant difference in serum TNF-α between the experimental CCI groups (11.84 ± 0.62 pg/ml vs 12.08 ± 0.25 pg/ml; *p* = NS) versus the SHAM controls, except for the CCI + PGVG group versus SHAMs (15.21 ± 0.26 pg/ml vs 12.08 ± 0.25 pg/ml; *p* < 0.05). Serum IL-6 levels were significantly higher in the CCI + PGVG (25.52 ± 2.32 pg/ml; *p* < 0.01) and the CCI + CBD (26.03 ± 1.58 pg/ml; *p* < 0.01) groups compared to the IL-6 levels in SHAM controls (12.19 ± 0.98 pg/ml). The serum IL-6 levels in CCI (12.23 ± 0.71 pg/ml) and CCI + Air (13.17 ± 1.02 pg/ml) exposure controls were similar to the serum IL-6 levels in the SHAM group.

Similar to the protein level results from serum samples, mRNA for IL-6 and TNF-α were significantly increased in lung tissue samples from CCI-PGVG rats as compared to SHAM controls (Fig. 7C and D), further implicating PGVG as an immunostimulant.

### Oxidative stress in lung

**Fig. 8:**
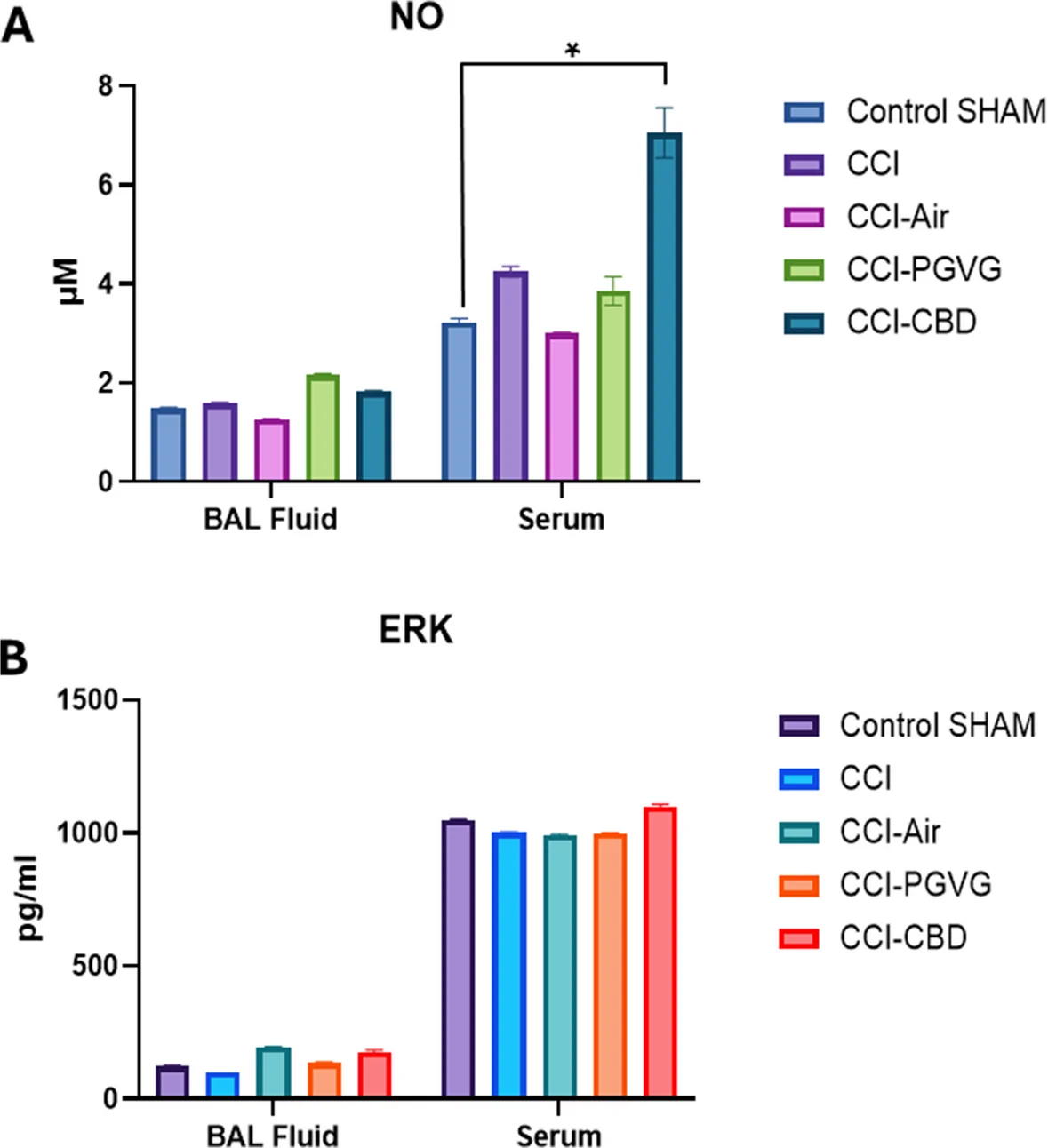
Quantification of NO and phosphor ERK-1 levels in serum and BAL fluid from all rat groups. **A** NO levels were quantified in serum and bronchoalveolar lavage (BAL) fluid from all rat groups using the Griess Assay. NO levels in serum were significantly higher than in BAL fluid (*p* < 0.002). NO levels were also significantly elevated in the CCI-PGVG and CCI + CBD groups compared to SHAM controls (*p < 0.05 and **p < 0.01, respectively). **B** Phospho ERK-1 levels were measured in serum and BAL fluid. While serum ERK levels were higher, no significant differences were observed between CCI groups and SHAM controls, reflecting a lack of significant CBD-induced changes in ERK phosphorylation

We observed that the NO levels were significantly higher in the serum [1.63 ± 0.12 μM (BAL) vs 3.94 ± 0.50 μM (serum) *p* < 0.002)] (μM) as compared to NO levels in the BAL fluid (Fig. 8A). Serum NO levels were significantly elevated in both the CCI-PGVG (3.56 ± 0.09 μM; *p* < 0.05) and CCI + CBD (6.08 ± 0.57 μM; *p* < 0.01) groups as compared to the SHAM control (3.07 ± 0.05 μM). NO levels in the CCI group were not significantly different when compared to NO levels in the SHAM controls (4.05 ± 0.093 μM vs 3.07 ± 0.05 μM; *p* = NS), respectively. We propose that NO accumulates in the lungs after the onset of exposure to CBD vapor. This results in iNOS expression in alveolar macrophages and nitrotyrosine formation in soluble serum proteins, which may contribute to increased oxidant stress mediated by NO-induced lung injury in people who vape CBD.

As shown in Fig. 8B, ERK levels are significantly higher in serum than in BAL fluid serum [332.3 ± 15.2 pg/ml (BAL) vs 843.1 ± 167.6 pg/ml (serum) *p* < 0.054)]. This mirrors that observed for NO levels, however no significant differences in serum ERK levels between CCI groups of animals and SHAM controls were observed. CBD receptors are known to transactivate multiple receptor tyrosine kinases and regulate serine/threonine kinases to activate ERK, and CBD-induced ERK phosphorylation occurs in phases over a short time-period of a couple of minutes (Dalton and Howlett 2012). Our study involved serum and BAL collection as an end point collection at animal sacrifice, we were not able to capture significant changes in ERK phosphorylation between study groups.

**Fig. 9:**
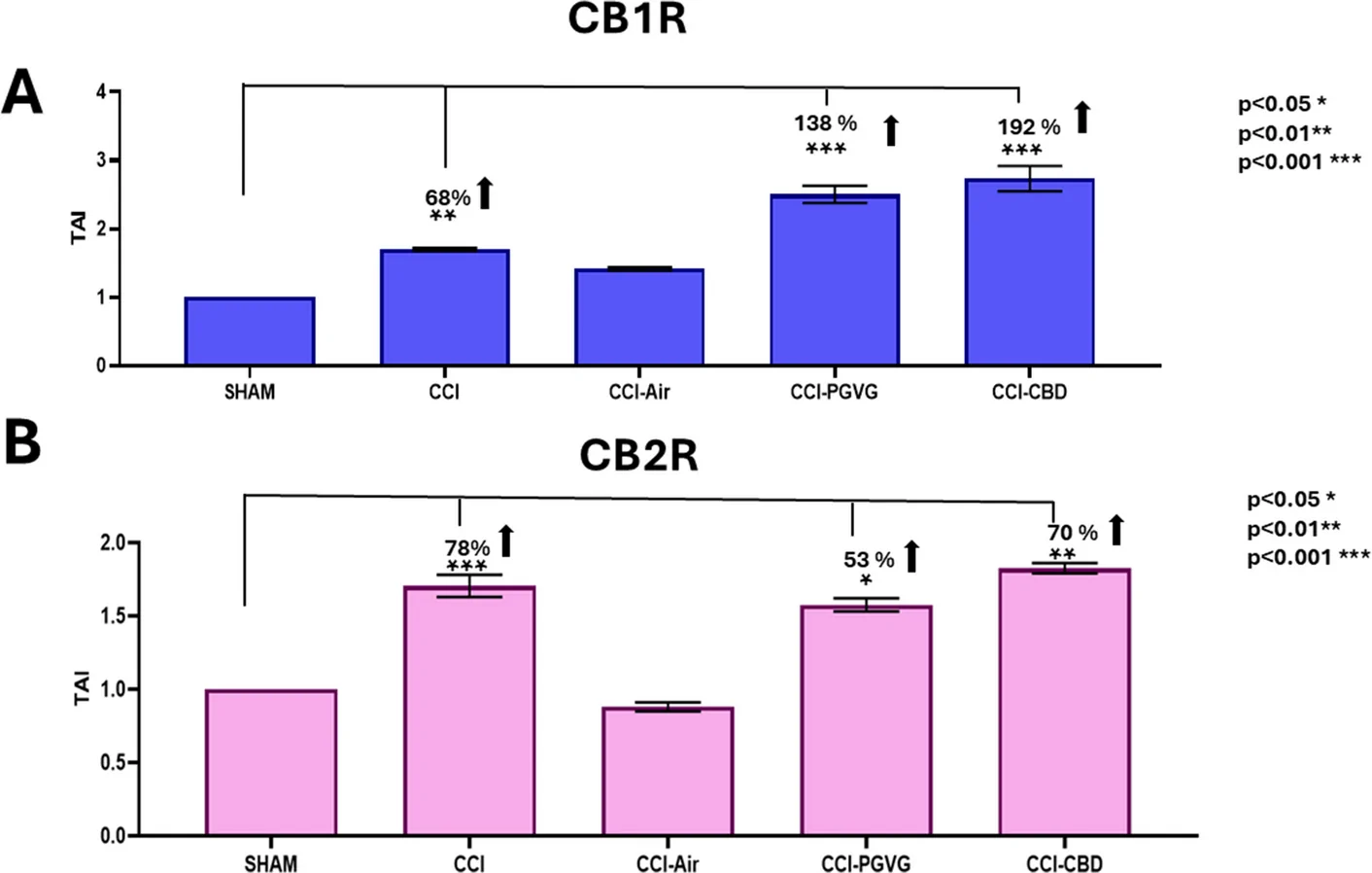
CBD receptor gene expression levels in lung tissue from all rat groups. **A** CB1R: CB1 receptor (CB1R) gene expression was significantly upregulated in lung tissue from CCI rats compared to SHAM controls (68% increase, **p < 0.01), with further increases observed in the CCI + PGVG and CCI + CBD groups following exposure to CBD vapor. Significant increases were noted at different time points and exposure levels, reflecting the impact of vaporized compounds on CB1R expression. **B** CB2R: CB2 receptor (CB2R) gene expression also showed a significant upregulation in CCI rats compared to SHAM controls (78% increase, ***p < 0.001). Both CCI + PGVG and CCI + CBD groups exhibited additional increases in CB2R expression, indicating that exposure to vaporized compounds, particularly CBD, led to further receptor upregulation. Error bars are plotted as ± SEM to indicate variability within the groups

The Cannabinoid Receptor 1 (CB1R) mediates most of the psychoactive effects of cannabinoids, whereas the Cannabinoid Receptor 2 (CB2R) is involved in anti-inflammatory and immunosuppressive actions (Bozkurt 2019). Our gene expression data shows that both CB1R and CB2R are present on lung tissue and that there is a significant upregulation of gene expression for both of these CBD receptors in the CCI group (68% increase for CB1R, *p* < 0.01; and 78% increase for CB2R, *p* < 0.001) compared to the respective SHAM controls, indicating that neuropathic pain induced increased expression of these receptors in the lung (Fig. 9). We also observed increased expression of CB1R and CB2R in CCI animals exposed to PGVG vapor (138% increase for CB1R, *p* < 0.001; and 53% increase for CB2R, *p* < 0.05) as well as in those exposed to CBD vapor (192% increase for CB1R, *p* < 0.001; and 70% increase for CB2R, *p* < 0.01). This corroborates reports that CNS injury can induce the upregulation of CB1R and CB2R expression (Ashton et al. 2006).

### CBD and inflammasome activation

**Fig. 10:**
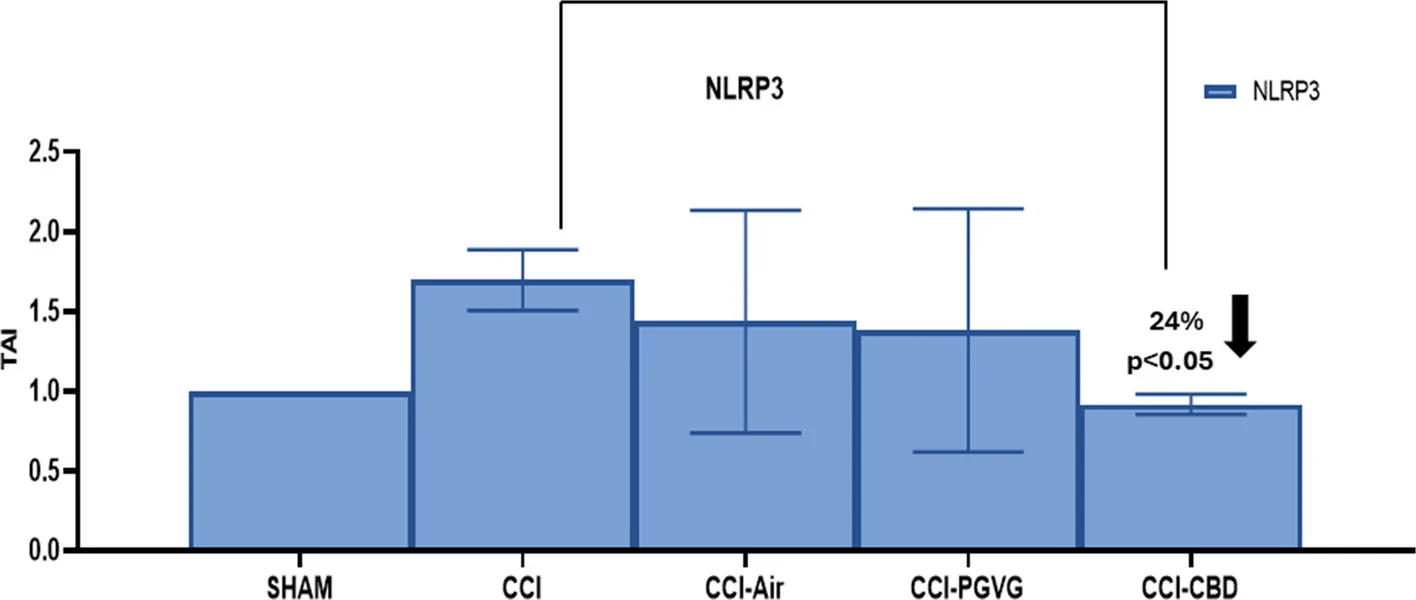
NLRP3 inflammasome expression levels in lung tissue from all rat groups. A 68% increase (**p < 0.01) was observed in the CCI group compared to SHAM controls, while PGVG exposure caused a 44% increase (*p < 0.05). CBD vapor exposure in CCI rats resulted in a decrease in NLRP3 expression (24% reduction, *p* = 0.057), indicating potential anti-inflammatory effects of CBD vapor

We observed a decrease (24% decrease; *p* < 0.057) in NLRP3 gene expression in CCI animals exposed to CBD vapor as compared to CCI alone (Fig. 10). We did not observe a significant difference between CCI animals exposed to CBD and the SHAM controls. CCI injury resulted in a 68% increase (*p* < 0.01) in NLRP3 gene expression, while PGVG exposure resulted in a 44% increase (*p* < 0.05) as compared to the SHAM controls, indicating that a pain stimulus and exposure to PGVG activated the NLRP3 inflammasome pathway. CBD vapor exposure in the CCI animals appears to result in an anti-inflammatory response as indicated by a decrease in NLRP3 expression.

## Discussion

The popularity of Cannabis and CBD vaping, especially in e-cigarettes, raises concerns about lung health. While Cannabis vaping has been linked to chronic lung inflammation and pathophysiological changes, CBD vaping is not fully understood. This study shows the in vivo effects of CBD vaping, focusing on lung pathology and analgesic efficacy. Thermal hyperalgesia was assessed only to confirm CCI-induced neuropathic pain and as described previously in the Methods, was not repeated during CBD treatment to avoid repeated heat sensitization or stress related variability that could confound behavioral outcomes. Consequently, treatment-related effects on thermal sensitivity were not evaluated. Instead, mechanical allodynia and hyperalgesia were the primary behavioral endpoints, as they provide more stable measures of chronic neuropathic pain and its modulation by therapeutic interventions. Cold allodynia was included as a supportive modality to assess peripheral pain sensitivity, though the dataset was limited by smaller group sizes. Mechanical allodynia was observed in CCI rats, indicating a lowered pain threshold to innocuous mechanical stimuli. We recently reported the analgesic effects of systemically (i.p.) administered CBD and extended efficacy using a novel CBD nanoformulation in CCI rats (Qayum et al. 2024). In the current study, CCI rats exposed to CBD vapor also exhibited reduced allodynia, with greater analgesic effects after two weeks exposure. Additionally, CBD vaporing decreased mechanical hyperalgesia, with similar efficacy after one and two weeks. These results indicate a significant impact of CBD treatment on CCI-induced mechanical allodynia and hyperalgesia.

Cold allodynia was evaluated by acetone application to the hind paws. CBD vapor did not alleviate cold-induced pain, indicating no efficacy in this pain modality. In a recent study, female rats were exposed to high-CBD vapor (> 2.5 mg CBD per 5-s puff) twice daily for 20 days, with 5-s puffs delivered every 30 s in 30-min sessions (Rivera-Garcia et al. 2023). Their findings showed a moderately attenuated neuropathy-induced cold allodynia, suggesting potential differential responses to CBD vapor across pain modalities. Our study used a lower CBD dose (0.27 mg per 55 mL puff) with a shorter exposure duration (30 min, 3-s puffs, 30-s intervals for 10 days). This difference may explain the lack of effect on cold allodynia. We standardized the exposure topography with a 55 mL puff volume, 3-s puff duration, and 30-s intervals, testing e-liquids containing carrier solvents (PG/VG 50/50) and 25 mg/mL CBD, resulting in a TPM concentration of 0.27 ± 0.05 mg/puff for 6 h/day over 21 days. Controls were exposed to filtered air. Their use of female rats contrasts with our male rats, complicating conclusions regarding dose, exposure duration, or gender-specific responses. Importantly, while mechanical stimuli are centrally mediated, cold pain is peripherally mediated (Mckemy 2018; Meyer et al. 2006; National Research Council, 2009), suggesting a central analgesic mechanism for CBD vapor, as evidenced in Figs. 3, 4, 5, 6 and 7. These findings enhance our understanding of CBD's risks and benefits for pain management, highlighting the need for further research to elucidate CBD's effects on pain perception across different modalities.

Histopathological analysis of lung tissue revealed significant histological alterations, especially in CCI rats treated with CBD vapor, providing crucial insights of chronic CBD vapor exposure on lung tissue integrity. Thickened alveolar walls, disruptions in the alveolar-capillary barrier, and enlarged alveoli were notable features observed in CCI + CBD rats. The presence of RBCs, macrophages, and hyaline material within some alveoli further underscores the complexity of lung injury associated with CBD vapor exposure. These histopathological changes suggest a multifaceted mechanism underlying CBD-induced lung injury. The thickened alveolar walls may indicate fibrotic changes and/or inflammation, triggered by prolonged exposure to CBD vapor. Disruptions in the alveolar-capillary barrier could result from increased vascular permeability and endothelial damage, leading to hemorrhage and impaired gas exchange (Bhattacharya & Matthay, 2013). Enlarged alveoli may reflect alveolar dilation and air trapping, indicative of emphysematous changes. The presence of RBCs and macrophages within alveoli suggests ongoing inflammation and tissue damage, likely due to inflammatory mediator release. The accumulation of hyaline material suggests proteinaceous fluid leakage into alveolar spaces, contributing to impaired lung function and respiratory compromise. In contrast, control rats subjected to CCI surgery showed only mild lung injury, with minor RBC and protein leakage, indicating that the extent of lung damage was significantly greater in CCI + CBD-exposed rats. However, the extent and severity of histopathological alterations were less pronounced in rats subjected to CCI surgery alone compared to those exposed to CBD vapor, suggesting a potential exacerbating effect of CBD on lung pathology. Sham-operated rats, which underwent surgical procedures without nerve injury, predominantly displayed normal lung morphology, highlighting the specificity of histopathological changes associated with neuropathic pain and CBD vapor exposure.

In addition to the structural pathology, cytokine analysis revealed elevated IL-6 and TNF-α levels in both BAL fluid and lung tissue, implicating PGVG vapor as a driver of pulmonary inflammation. IL-6, a key mediator that amplifies immune signaling through NLRP3 inflammasome activation (Xiao et al. 2022), provides a mechanistic link between vapor exposure and lung inflammatory responses. The elevated IL-6 observed in CCI + PGVG and CCI + CBD groups suggests that while CBD vapor may offer analgesic benefits, it may concurrently promote inflammatory signaling in the lung, potentially disrupting the balance between pro- and anti-inflammatory cytokines. These findings align with our observed histopathological changes and support a dual effect of CBD vapor, anti-nociceptive yet pro-inflammatory in pulmonary tissue. This duality is consistent with the context-dependent pharmacology of CBD. While CBD exerts anti-inflammatory and cytoprotective actions in many systemic or neural models—primarily through CB2, TRPV1, adenosine, and PPARγ signaling—it can produce pro-inflammatory outcomes in epithelial tissues exposed to high local concentrations or oxidative conditions. Aerosolized CBD undergoes thermal and oxidative transformation during vaporization, generating reactive intermediates capable of triggering epithelial stress responses and NLRP3 inflammasome activation. In this framework, the up-regulation of IL-6 and TNF-α observed here likely reflects a route- and tissue-specific inflammatory response, rather than a contradiction of CBD’s canonical anti-inflammatory profile. Hence, CBD’s immunomodulatory effects should be viewed as bi-phasic and microenvironment-dependent, with inhalation exposure representing a unique pulmonary context where oxidative stress outweighs its systemic anti-inflammatory potential.

Our findings align with reports of diffuse alveolar damage, bilateral ground-glass opacities, and increased inflammatory cells in BAL of E-cigarette or Vaping Use-Associated Lung Injury (EVALI) patients (Marrocco et al. 2022), highlighting similarities induced by inhalation exposure. Additionally, mice exposed to vitamin-E-acetate (VEA), medium-chain triglycerides, or CBD e-cigarette vapors for 3 to 28 days exhibited increased inflammatory markers and disruptions in the alveolar-capillary barrier. The presence of VEA in BAL was observed only in VEA-treated mice, accompanied by progressive increases in BAL total fluid volume, total cell count, and total protein, with the presence of large vacuolated macrophages. Lung parenchymal alterations, including alveolar and parenchymal inflammation, monocyte and neutrophil influx, foamy macrophages, alveolar septal thickening, and lymphocyte-rich aggregates around blood vessels, were observed (Bhat et al. 2020; Matsumoto et al. 2020; Muthumalage et al. 2020). The mechanistic basis for cannabis-induced lung inflammation and hyperinflammation remains unclear. CB1R and CB2R play critical roles in respiratory homeostasis and lung functionality, with CBD's role in immune response and pain modulation remaining complex. While CBD may be therapeutic by inhibiting immune function, it may also impair lung function and slow respiratory pathogen clearance (Turcotte et al. 2016). CB2Rs are implicated in neurogenic inflammation, acting through sensory nerves (Bozkurt 2019), and both receptors help maintain respiratory balance (Calignano et al. 2000; Niederhoffer et al. 2003; Rice et al. 1997; Schmid et al. 2003). CB1Rs mediate most psychoactive effects, while CB2Rs play a role in anti-inflammatory and immunosuppressive actions (Cabral et al. 2008). CBD aerosols induce greater lung inflammation, higher oxidative stress, and disrupts lung epithelial barrier (Bhat et al. 2023). Literature on CBD's effect on inflammasome activation is limited. CBD treatment induced an inactive state of the NLRP3 inflammasome, limiting inflammation and promoting survival, with nuclear factor kappa B (NF-κB) signaling involved in NLRP3 priming (Libro et al. 2016). Our findings show a reduced inflammatory response in CCI animals exposed to CBD vapor (Figs. 7, 8 and 10), suggesting that the pain relief results from CBD's inhibitory effects on inflammasomes and downstream proteins. This supports the potential of CBD as an inflammasome-inhibitory drug target. The histopathological changes in this study highlight the potential adverse effects of chronic CBD vapor exposure on lung tissue integrity and function. Further research is needed to identify the mechanisms driving CBD-induced lung injury and to assess long-term effects on respiratory health. Such studies are critical for shaping public health policies and clinical guidelines, particularly for individuals with pre-existing lung conditions or at risk of respiratory complications.

While our study provides valuable preliminary insight into the risks and potential mechanisms of CBD vaping, the work was conducted as a pilot, proof-of-concept investigation designed to establish feasibility and generate foundational data. Given the bidirectional inflammatory potential of CBD depending on dose and route, these findings should be interpreted cautiously. Accordingly, the findings are exploratory and should be interpreted as hypothesis-generating.

### Limitations

This investigation was conducted as a pilot, proof-of-concept study designed to establish feasibility and generate preliminary data on the pulmonary and analgesic effects of CBD vapor exposure. While the findings offer valuable mechanistic insights, several limitations must be acknowledged.

The overall sample size was small, reflecting the feasibility-focused design and the ethical and logistical constraints associated with multi-endpoint sampling in a single protocol. Although this limits statistical power and formal generalization, the reproducibility of trends across independent outcome domains—behavioral, histopathological, and molecular—supports the robustness of the underlying biological signal.

Only male rats were used to minimize variability, but this restricts the ability to infer potential sex-dependent differences in CBD’s effects. Future studies incorporating female cohorts will be essential to explore potential sex-specific responses in both pain modulation and pulmonary outcomes.

The exposure paradigm was limited to a two-week duration, representing an acute-to-sub-chronic exposure model. Longer exposure regimens will be needed to assess chronic pulmonary effects and recovery potential following CBD vapor inhalation. Additionally, the current design did not evaluate thermal hyperalgesia during vapor exposure, as this assessment was restricted to confirming neuropathy post-CCI surgery, consistent with prior studies focusing on mechanical and cold modalities.

Furthermore, thermal hyperalgesia was not reassessed during vapor exposure to prevent repeated heat sensitization, a methodological decision consistent with prior neuropathic pain studies emphasizing mechanical endpoints as more reliable chronic pain indicators. In addition, lung histopathology was evaluated descriptively without numerical scoring. While this limits quantitative comparison across groups, the consistent presence of key pathological features across all exposed animals, together with concordant molecular evidence of inflammasome activation, supports the biological validity of the observed pulmonary effects. Likewise, we acknowledge that the PGVG vehicle control itself elicited measurable inflammatory responses, consistent with previous reports of humectant-induced pulmonary irritation. This overlap makes it challenging to fully isolate CBD-specific effects from those arising due to carrier solvents. However, the inclusion of the PGVG-only (vehicle) exposure group allowed relative differentiation of CBD-related outcomes from baseline vapor effects, and future studies will incorporate refined exposure matrices to better delineate these interactions. Despite these constraints, the study provides important foundational evidence supporting the feasibility and biological relevance of a rodent CBD vapor inhalation model. These results should be interpreted as hypothesis-generating and serve to inform the design of larger-scale, sex-balanced, and longitudinal studies aimed at elucidating the full risk–benefit profile of CBD vapor use.

## Conclusion

Our study underscores the importance of assessing the safety profile of CBD vaping, particularly in the context of chronic pain management. If cannabis is used for therapeutic benefits, it is essential to uncover the mechanisms that underlie the impact cannabis can have on lung pathophysiology. Gene and protein expression analysis using qPCR and ELISA highlights the role of the NLRP3 inflammasome, key cytokines, as well as modulation of CB receptor expression in CBD-induced lung inflammation and neuropathic pain.

Our findings reveal the dual nature of CBD vapor as a treatment modality for sciatic nerve injury-induced chronic pain and its potential risk to lung health. It is important to note that, under the present experimental conditions, the observed pulmonary toxicity appeared to outweigh the limited analgesic benefits. Although CBD vapor exposure effectively mitigated mechanical hyperalgesia linked to the CNS, it failed to alleviate cold allodynia associated with the PNS. This dichotomy underscores a partial efficacy in neuropathic pain relief. More alarmingly, CBD vapor induced substantial lung pathology. These pulmonary effects suggest a concerning biological signal, indicating that CBD vapor may pose substantial risk to lung health if used chronically. Hence, this dual effect necessitates a cautious evaluation of CBD vapor use.

Although CBD vaping has promising effects in alleviating pain, the variable modulation of CBD-induced inflammation response and the potential airway responsiveness following CBD treatment may be attributed to its affinity to multiple receptors. CBD interacts with receptors including the Transient Receptor Potential Cation Channels (TRP), serotonin receptor 1 A (5-HT1A), adenosine receptors A1 and A2, Gamma-aminobutyric acid (GABAA) receptors and nuclear receptors such as the Peroxisome Proliferator-Activated Receptor (PPARγ), thereby modulating proinflammatory mediators. CBD also modifies membrane and organelle calcium channels, altering intracellular signaling (Martinez et al. 2023; Morales et al. 2017).

Future investigations should prioritize expanded cohort sizes, extended exposure durations, and thorough molecular profiling to yield definitive insights into the risks and therapeutic benefits of CBD vapor inhalation. Further, a detailed evaluation of the NLRP3 inflammasome pathway needs to be undertaken with a focus on NF-κB signaling in the context of NLRP3 priming. These studies are pivotal for shaping regulatory guidelines and clinical strategies pertaining to CBD-based therapies, particularly among susceptible cohorts with respiratory or pain-related comorbidities. Implementing such rigorous methodologies is essential to fully exploit CBD's therapeutic efficacy.

## Data Availability

The datasets used and/or analysed during the current study are available from the corresponding author on reasonable request.

## Abbreviations

BAL: Bronchoalveolar lavage
BL: Baseline
CB1R: Cannabinoid receptor 1
CB2R: Cannabinoid receptor 2
CBD: Cannabidiol
CCI: Chronic constriction injury
EVALI: E-cigarette use-associated lung injury
IASP: International Association for the Study of Pain
LAF: Laboratory animal facility
NBF: Neutral buffered formalin
NF-κB: Nuclear factor kappa B
NO: Nitric oxide
NLRP3: NOD-like receptor family, pyrin domain-containing 3
PG: Propylene glycol
PGVG: Propylene glycol and vegetable glycerin
PWL: Paw withdrawal latency
TAI: Transcript accumulation index
THC: Tetrahydrocannabinol
TPM: Total particulate matter
VEA: Vitamin-E-acetate
VG: Vegetable glycerin
